# Supermassive Dark Star candidates seen by JWST

**DOI:** 10.1073/pnas.2305762120

**Published:** 2023-07-11

**Authors:** Cosmin Ilie, Jillian Paulin, Katherine Freese

**Affiliations:** ^a^Department of Physics and Astronomy, Colgate University, Hamilton, NY 13346; ^b^Weinberg Institute for Theoretical Physics, Texas Center for Cosmology and Astroparticle Physics, Department of Physics, University of Texas, Austin, TX 78712; ^c^Department of Physics, Stockholm University, Stockholm SE-106 91, Sweden; ^d^Nordic Institute for Theoretical Physics (NORDITA), Stockholm SE-106 91, Sweden

**Keywords:** cosmology, dark matter, first stars

## Abstract

The first generation of stars in the universe is yet to be observed. There are two leading theories for those objects that mark the beginning of the cosmic dawn: hydrogen burning Population III stars and Dark Stars, made of hydrogen and helium but powered by dark matter heating. The latter can grow to become supermassive (*M*_⋆_ ∼ 10^6^*M*_⊙_) and extremely bright (*L* ∼ 10^9^*L*_⊙_). We show that each of the following three objects—JADES-GS-z13-0, JADES-GS-z12-0, and JADES-GS-z11-0 (at redshifts *z* ∈ [11, 14])—are consistent with a Supermassive Dark Star interpretation, thus identifying the first Dark Star candidates.

## Significance

In 2007, we proposed the idea of Dark Stars. The first phase of stellar evolution in the history of the universe may be Dark Stars (DS), powered by dark matter (DM) heating rather than by nuclear fusion. Although made almost entirely of hydrogen and helium from the Big Bang, they form at the centers of protogalaxies where there is a sufficient abundance of DM to serve as their heat source. They are very bright diffuse puffy objects and grow to be very massive. In fact, they can grow up to ten million solar masses with up to ten billion solar luminosities. In this paper, we show that the James Webb Space Telescope may have already discovered these objects.

The James Webb Space Telescope (JWST) is poised to revolutionize our understanding of the formation and properties of first luminous objects in the universe. Since beginning to take data, JWST has discovered a surprising number of extremely bright high redshift galaxy candidates (e.g., refs. 1–5), which are difficult to reconcile with expectations from numerical simulations of the universe in the canonical *Λ*CDM scenario. Moreover, ref. 6 shows that, in view of HST data, it is unlikely that any *Λ*CDM modifications can be viable solutions to this tension. In this paper, we propose a different avenue and show that some of the JWST high redshift galaxy candidates could instead be Dark Stars (DSs), early stars made almost entirely of hydrogen and helium but powered by dark matter (DM) heating rather than by fusion. Dark Stars provide a good match to JWST data, both in terms of their spectra (good fits to JWST photometry) and in that JWST (given its angular resolution) cannot rule out a point-source interpretation of many of these candidates.

Prior to JWST, we had very limited data on the cosmic dawn era, i.e., the period when the first stars and galaxies form. As such, numerical simulations were the primary tool to describe the properties of the first stars (e.g., refs. 7–13), and galaxies (e.g., refs. 14–19) in the universe. In the standard picture of the first (Population III a.k.a. Pop III) stars, they formed roughly 100–400 Myrs after the Big Bang (*z*∼20–10) as a consequence of the gravitational collapse of pristine, zero metallicity, molecular hydrogen clouds at the center of 10^6^–10^8^*M*_⊙_ minihaloes. Pop III stars grow via accretion, reaching masses of (at most) 10^3^*M*_⊙_ (e.g., ref. 20), and populate the first galaxies. We previously proposed, however, a different type of first star: Dark Stars (21–23), early stars powered by DM heating rather than by fusion.

Some of the JWST high redshift galaxy candidates could instead be Dark Stars, which are made almost entirely of hydrogen and helium with less than 0.1% of the mass in the form of DM. Since they remain cool (without a central hot core), there is no fusion inside them; instead, DM annihilations happen throughout their volume. Dark Stars are giant, puffy (∼10 AU), and cool (surface temperatures ∼ 10,000 K) objects. We follow the evolution of Dark Stars, in thermal and hydrostatic equilibrium, from their inception at ∼1*M*_⊙_ as they accrete mass from their surroundings to become Supermassive Dark Stars (SMDSs), some even reaching masses > 10^6^ *M*_⊙_ and luminosities > 10^10^ *L*_⊙_, making them visible to JWST (24, 25). Once the DM runs out and the SMDS dies, it collapses to a black hole; thus, Dark Stars may provide seeds for the supermassive black holes observed throughout the universe and at early times (see, e.g., ref. 26 for statistical studies of such supermassive black holes, inferred from properties of high redshift quasars).

As of this writing, out of the hundreds of potentially high-*z* objects discovered by JWST, only ∼10 have been confirmed spectroscopically via the identification of the Lyman-break feature in their spectral energy distributions (SEDs). In particular, the JWST Advanced Deep Extragalactic Survey (JADES) has discovered four spectroscopically confirmed Lyman break objects: JADES-GS-z13-0, JADES-GS-z12-0, JADES-GS-z12-0, and JADES-GS-z10-0 (27, 28). In this paper, we will show that three of these four JADES high-*z* objects are consistent with SMDs. We find that, with the exception of JADES-GS-z10-0, the photometry of those objects can be modeled by SMDSs Spectra.

For a more definitive answer, higher-quality spectroscopy of the objects will be required. Specifically, as we will show below, the appropriate helium lines could be smoking guns for Dark Stars and could distinguish between, for instance, a galaxy made of Pop III stars and a single SMDS. A helium-II absorption feature at 1640 Å would be characteristic of a hot SMDS (25). An emission line at the same wavelength would be characteristic of a Pop III/II galaxy. For all types of SMDSs, the Balmer absorption lines, at rest frame wavelengths *λ* ≳ 0.35 μm can be used to differentiate them from early galaxies, which will typically exhibit emission lines at the same wavelengths (29).

## Dark Stars

As the molecular clouds of hydrogen collapse inside early minihaloes in the process of star formation, the large reservoir of DM at the centers of the minihaloes can play an important role. If the DM particles are their own antiparticles, then their annihilation provides a heat source that stops the collapse of the clouds and in fact produces a different type of star, a Dark Star, in thermal and hydrostatic equilibrium. We wish to emphasize that Dark Stars are made almost entirely of ordinary matter (hydrogen and helium) but powered by DM, even though the DM only constitutes less than 0.1% of the DS. We considered two types of DM particles: weakly interacting DM (WIMPs, in most of our papers) and self-interacting DM (SIDM).^*^ The energy production per unit volume provided by the annihilation of two DM particles is given by:

[1] $$\begin{array}{c} Q=m_{\chi }n_{\chi }^{2}\left\langle \sigma v\right\rangle =\left\langle \sigma v\right\rangle \rho_{\chi }^{2}/m_{\chi }, \end{array}$$

where *m*_*χ*_∼ 1 GeV–10 TeV is the DM mass, *n*_*χ*_ is the DM number density, and *ρ*_*χ*_ is the DM energy density. We have used the fact that the DM mass is converted to energy in the annihilation, and we took the standard annihilation cross-section (the value that produces the correct DM abundance in the universe today): ⟨*σv*⟩=3 × 10^−26^cm^3^/s. We note that cross-sections several orders of magnitude smaller or larger would work equally well; by considering a variety of DM masses, we can see from Eq. **1** that this is equivalent to considering a variety of cross-sections. Three key ingredients are required for the formation of DSs: 1) sufficient DM density, 2) DM annihilation products become trapped inside the star, and 3) the DM heating rate beats the cooling rate of the collapsing cloud. In our previous work, we showed that all three criteria can be easily met (21).

The criterion of high DM density can be met in two ways. First, as the hydrogen cloud collapses, it dominates the potential well and pulls in more DM with it. This phenomenon can be well described by adiabatic contraction (AC).^†^ Since many DM particles are on chaotic or box orbits, the central DM density can be replenished and kept high for millions (to billions) of years. Second, once the DM power is depleted, the star starts to collapse and, in the process, reaches a high enough density that it is able to capture further DM particles via elastic scattering of the DM with the atoms in the star. We will consider both extended AC and capture in this paper. For the latter case, we assume the following value for the product between the DM ambient density at the location of the star and the elastic scattering cross-section: *ρ*_*χ*_*σ* = 10^14^ GeVcm^−3^ × 10^−40^cm^2^. The DM density assumed is consistent with estimates based on the adiabatic contraction prescription (21, 32) and the scattering cross-section is within the allowed region of parameter space for spin-dependent interactions (33, 34).

Once a DS forms of ∼1*M*_⊙_, we have studied its evolution with two different types of stellar codes: one of which assumes that the DS can be approximated as a polytrope and the MESA stellar evolution code (35). In both cases, we find essentially the same results (36). Because the DS are puffy and cool (surface temperatures ∼ 10^4^K), they are able to accrete the material around them and become very massive (there is not enough ionizing radiation to prevent accretion). We find the equilibrium structure for the stars of a given mass and then build up the stars one solar mass at a time, always in equilibrium, and find that some of them can become SMDS that are incredibly massive (> 10^6^*M*_⊙_) and bright (> 10^9^*L*_⊙_), and the heaviest ones should be visible in JWST. Because they are simultaneously bright, they may look different from competing objects.

## Method

We describe below our method to look for Dark Star candidates in JWST data. High redshift luminous objects are typically discovered as photometric dropouts in deep field surveys.^‡^ Our focus in this paper is dropout candidates identified already in the literature that satisfy the following two criteria: i) a Lyman break has been spectroscopically identified, such that the objects are definitively at high (*z* ≳ 10) redshift,^§^ and ii) the objects are unresolved, or marginally resolved, so that they can be consistent with an explanation in terms of a point object. Any object that satisfies both criteria can be consistent with the hypothesis that it is a Dark Star.

We have found three candidates that match both criteria. Specifically, of the four spectroscopically confirmed Lyman break objects—JADES-GS-z13-0, JADES-GS-z12-0, JADES-GS-z11-0, and JADES-GS-z10-0 (27, 28)—all are consistent with possibly being point objects, and three have photometry that can be modeled by SMDS spectra (with the exception of JADES-GS-z10-0).

*Resolved vs unresolved objects:* SMDSs would be point objects in JWST data, whereas galaxies are larger and hence may be resolved. The angular resolution of JWST is approximately *θ*_*res*_ ∼ 10^−6^ radians (37). At *z* ≃ 10, a SMDS (with radius *R* ∼ 10 AU) will have an unlensed angular size of ∼10^−13^ radians, well below the angular resolution of any imaginable telescope. Even if strongly lensed, SMDSs will still be below the resolution limit of JWST, i.e., look like point sources. Some of the galaxy candidates identified in the JWST data are barely resolved, or unresolved, i.e., consistent with point sources.

The four JADES objects we are considering are all consistent with a point-source interpretation. For two of them, JADES-GS-z11-0 and JADES-GS-z12-0, the authors argue that they are resolved, under the assumption of an interpretation in terms of galaxies (27). However, their estimated effective sizes (∼0.02″ and ∼ 0.04″, approximately the size of one NIRCam pixel) are about one order of magnitude below the resolution limit of JWST NIRCam (∼0.1″). We therefore consider here the possibility that they are unresolved, point sources. We note that Airy patterns^¶^ would definitively identify them as point objects. However, since the first ring is only 1.75% as bright as the central spot,^#^ JWST would have to observe the object with an extremely long exposure time, O(year), and hence, discovery of such a pattern in JWST is unlikely. Yet, it is a signature of SMDS that future telescopes would be able to see.

*Dark Star Spectra:* Our next task is to show that the spectra of three of the four objects are well fit by Dark Star spectra. We note here that the spectra for those four objects, obtained in ref. 28, do not yet confidently identify any spectral lines, as they are too noisy (S/N ∼ 2). Follow-up spectroscopy is required in order to determine the presence of emission/absorption features. For this reason, we restrict our discussion in this paper to comparing SMDSs to photometric data (i.e., average fluxes in observed wavelength bands).

The spectra of SMDSs were obtained using the TLUSTY (39) synthetic stellar atmospheres code. This code accounts for not only the blackbody radiation from the photosphere of the DS but also for absorption or emission features (lines and breaks) arising from the gas in the atmosphere of the star. For each SMDS formation mechanism, we initially generated TLUSTY SEDs on a coarse stellar mass grid: ∼10^4^, 10^5^, 10^6^, 10^7^ (in units of *M*_⊙_) (25). If needed, in order to get an optimal fit, we further refined the grid to include midpoints. The aim of this paper is not to find the best possible SMDS fit to a given set of JWST photometric data but rather to stress that SMDSs are generically very good models to JWST photometric data for Lyman-break objects. Making the grid too refined would only diminish the impact of this statement, as it could seem that only under very special conditions one would find a SMDS to fit data. In fact, as we will show, we find that generically, they would fit well. Of course, once we get to the point of extracting DM parameters, from matching high-quality spectra obtained with JWST (or other observatories) with simulated SEDs of Dark Stars, we will need a very fine stellar mass grid, one which we are in the process of generating TULSTY spectra for. Moreover, in this study, we take DM mass *m*_*χ*_ = 100 GeV in obtaining SMDS spectra. In future work, we plan to consider a variety of other DM particle masses. Lighter (heavier) DM particles produce more (less) heat (see Eq. **1** and thus somewhat hotter (cooler) Dark Stars. Hence, the consideration of a variety of particle masses would increase the likelihood of finding SMDS spectra that are good photometric fits to JWST data. Below we discuss the main features of the SMDS SEDs (see also *SI Appendix*). For the SMDSs formed via DM capture, we find that the SEDs are nearly independent of the stellar mass since their temperatures are roughly constant. They also have a much steeper slope of the (UV) continuum when compared to the cooler SMDS formed via AC.

An important tool in the future to differentiate SMDSs from early galaxies will be the He II *λ*1640 absorption line at 0.1640 μm (restframe), present for all SMDSs formed via DM capture and for the *M*_⋆_ ≳ 5 × 10^5^*M*_⊙_ SMDSs formed via AC. In contrast, early galaxies would instead exhibit a nebular emission line at the same wavelength. For all types of SMDSs, the H Balmer absorption lines at rest frame wavelengths *λ* ≳ 0.35 μm can also be used to differentiate SMDSs from early galaxies, which will typically exhibit strong nebular Balmer emission lines instead.

In order to convert the rest frame SEDs of each SMDS considered into observable quantities, we first redshift them:

[2] $$\begin{array}{c} F_{\nu }\left(\lambda_{\mathrm{obs}};M_{⋆};z_{\mathrm{emi}}\right)=\frac{\left(1+z_{\mathrm{emi}}\right)4\pi R_{*}^{2}F_{\nu }\left(\lambda_{\mathrm{emi}};M_{⋆}\right)}{4\pi D_{L}^{2}\left(z_{\mathrm{emi}}\right)}, \end{array}$$

where *λ*_*obs*_ = (1 + *z*_*emi*_)*λ*_*emi*_ and *λ*_*emi*_ represent the observed and emitted wavelengths, R_*_ is the radius of the SMDS, *M*_⋆_ is its mass, and *D*_L_ is the luminosity distance. *F*_*ν*_(*λ*_*emi*_;*M*_⋆_) represents the rest frame flux density (plotted in *SI Appendix*), and *F*_*ν*_(*λ*_*obs*_; *M*_⋆_; *z*_*emi*_) is the redshifted flux density. We account for the Gunn–Peterson trough (40) by suppressing all the flux short-ward of the redshifted Ly-α line to zero, since all of the candidates we will be looking for are at *z*_*emi*_ ≳ 10.

*Comparing SMDS to JWST data:* In each JWST band (labeled here by the letter b), we find the average expected redshifted flux due to a SMDS:

[3] $$\begin{array}{c} {\tilde{F}}_{\nu ;b}\left(M_{⋆};z_{\mathrm{emi}}\right)=\frac{\int_{\lambda_{\min}}^{\lambda_{\max}}T\left(\lambda_{\mathrm{obs}}\right)F_{\nu }\left(\lambda_{\mathrm{obs}};M_{⋆};z_{\mathrm{emi}}\right)\frac{d\lambda_{\mathrm{obs}}}{\lambda_{\mathrm{obs}}}}{\int_{\lambda_{\min}}^{\lambda_{\max}}T\left(\lambda_{\mathrm{obs}}\right)\frac{d\lambda_{\mathrm{obs}}}{\lambda_{\mathrm{obs}}}}, \end{array}$$

where *λ*_*obs*_ is the observed wavelength, *T*(*λ*_*obs*_) is the throughput curve for the photometric band (filter) in question, and *λ*_*min*; *max*_ represents the two wavelengths defining the band-pass filter denoted here by the letter *b*.

*Best fit Dark Star models:* We then compare the predicted SMDS photometry on our stellar mass and formation mechanism grids to JWST data in all bands available for each candidate analyzed. Some of the JWST objects may be gravitationally lensed, which will enhance the flux by magnification factor *μ*. Therefore, we optimize the fit between model (*F*_*ν*; *b*_) and data (*f*_*ν*; *b*_) with regard to two main parameters: the redshift *z*_*emi*_ and *μ*. Using a *χ*^2^ analysis:

[4] $$\begin{array}{c} \chi^{2}=\sum_{b}\frac{(f_{\nu ;b}-\mu \times {\tilde{F}}_{\nu ;b}{\left(M_{⋆};z_{\mathrm{emi}}\right)}^{2}}{\sigma^{2}\left(f_{\nu ;b}\right)+\sigma_{\mathrm{sys}}^{2}\left(b\right)}, \end{array}$$

we can determine which combinations of *z*_*emi*_ and *μ* align most closely with the data. In Eq. **4**, we sum over all bands for which photometric data are available, and *σ*(*f*_*ν*; *b*_) represents the statistical flux error in a given band, whereas *σ*_*sys*_(*b*) represents the systematic error term, that accounts for zero point uncertainties or imperfect aperture corrections.

For each SMDS formation mechanism considered (AC or DM capture), we select the stellar mass in our TLUSTY grid for SMDS SEDs that will lead to a value of the *μ* parameter closest to 1, as the objects analyzed in this work are typically assumed to be unlensed. However, in reality, they could be lensed by *μ* as large as 𝒪(10). While there are no *known* foreground clusters or galaxies that could act as lenses for the objects considered here (JADES-GS-z11-0, JADES-GS-z12-0, and JADES-GS-z13-0), the fact that the objects are unresolved leaves open the possibility that they are indeed strongly lensed, with the resulting shear undetectable with current resolution. On the other hand, most lines-of-sight in the universe will have *μ* < 1 (with photons being pulled away from the line-of-sight toward nearby overdense regions) (41, 42). N-body simulations reveal that the probability of a given *μ* is a redshift-dependent function (*P*(*μ*; *z*)) for which the peak is moving toward lower values as *z*_*emi*_ is increased. For instance, at *z* = 3.2 the peak of *P*(*μ*) is around *μ* ≃ 0.9 (42), with values as low as *μ* ≃ 0.7 not ruled out. By *z* ∼ 10, even lower values of *μ* will not only be possible, but likely.

There is a degeneracy between *μ* and *M*_⋆_ with regard to the SMDSs models. For SMDSs formed via capture, this degeneracy is almost one to one, since the rest frame flux is largely independent of *M*_⋆_ (*Materials and Methods*). As such, the redshifted fluxes of SMDSs formed via capture, at any given wavelength, scale linearly with either *M*_⋆_ or *μ*. The degeneracy is still present for the SMDSs formed via AC, albeit less trivial, since now there is a stellar mass dependence of the rest frame fluxes. In summary, scanning over *μ* is a proxy for scanning over *M*_⋆_ while keeping *μ* fixed. In other words, for any optimal fit, we find for the three parameters—*μ*_*fit*_, *z*_*fit*_, and *M*_⋆_—there will be an equally good fit with *μ* ≈ 1, *z*_*fit*_, and a somewhat larger or smaller value of *M*_⋆_, depending on whether *μ*_*fit*_ is smaller or larger than one.

## Results

**Fig. 1.**
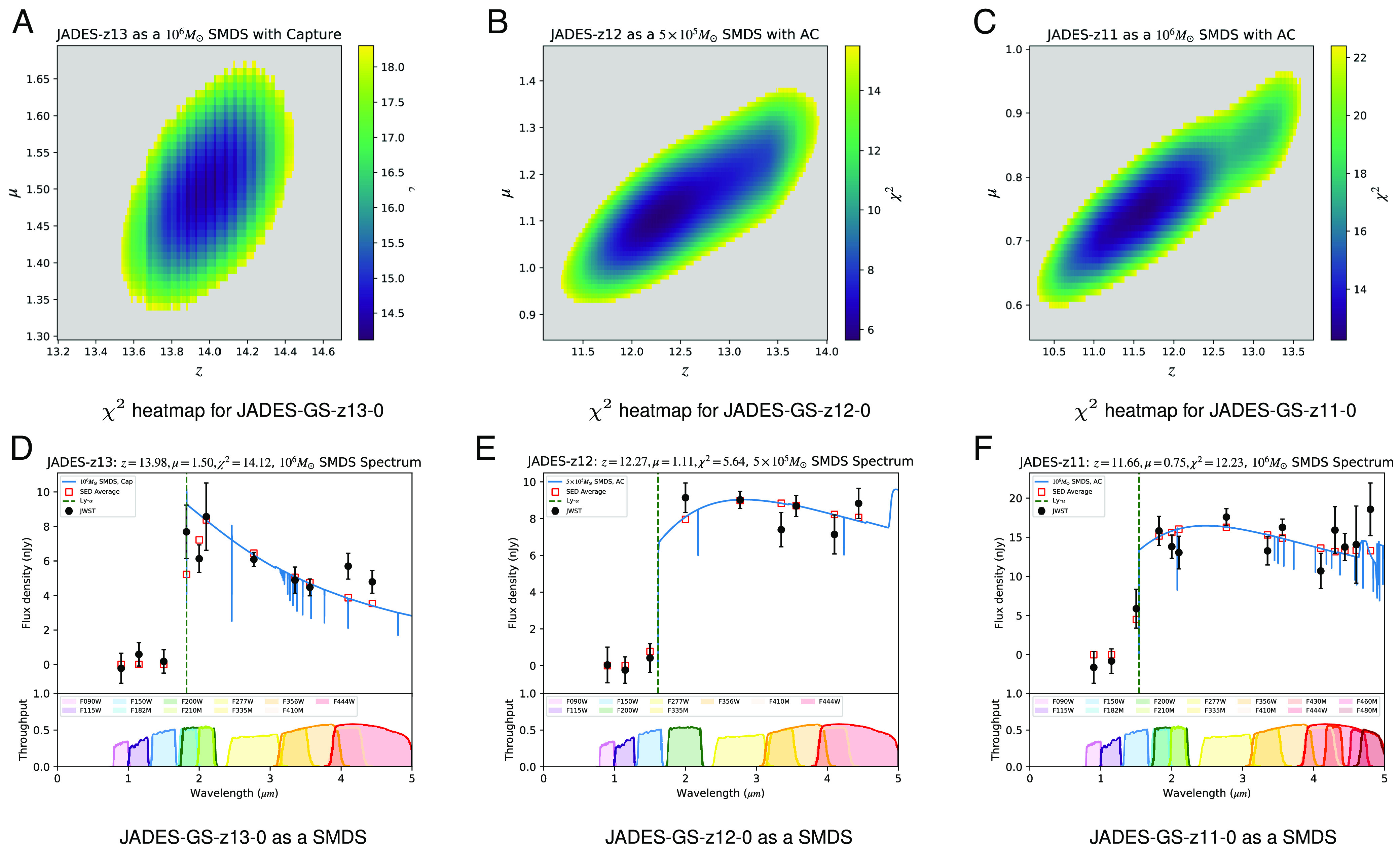
(*Top* Row: Panels *A*–*C*) Optimal fit regions in the *z* vs *μ* (magnification) parameter space for SMDS fits to JADES-GS-z11-0, JADES-GS-z12-0, and JADES-GS-z13-0 photometric data. The heatmap is color coded according to the value of the *χ*^2^ and is cutoff (grayed out) at the critical value corresponding to 95% CL. In addition to labeling the object, the title in each panel includes the mass and formation mechanism for the SMDSs model considered. (*Bottom* Row: Panels *D*–*F*) For each case, we plot our best fit SEDs against the photometric data of (27) in each band. Titles include values of relevant parameters and *χ*^2^. Each band is visually represented by its throughput curves, color coded and plotted at the bottom of the SED plots.

Three of the four JADES objects considered, JADES-GS-z13-0, JADES-GS-z12-0, and JADES-GS-z11-0, have photometry consistent at a minimum 95% confidence level (CL) with a Dark Star interpretation, as shown in Fig. 1. In the *Upper* panels, we have plotted the *χ*^2^ for the match in the *μ* vs *z* plane, where all points shown in the colored region fit the data at the 95% CL or better for each of the three objects as labeled.

### Best Fit Parameters.

For JADES-GS-z13-0 (*Left* panels), we find that a 10^6^*M*_⊙_ SMDS formed via DM capture, at *z*_*photo*_ ≃ 13.98,^ǁ^ boosted by a gravitational lensing factor of *μ* ≃ 1.5 leads to our best fit, with a *χ*^2^ ≃ 14.12. For this object, there are photometric data in 11 JWST bands, so that the 95% CL corresponds to the critical value *χ*_*crit*_^2^ ≃ 18.3. For JADES-GS-z12-0, our best fit is a 5 × 10^5^*M*_⊙_ SMDS formed via AC, at *z*_*photo*_ ≃ 12.27, lensed by a factor of *μ* ≃ 1.11, with a *χ*^2^ ≃ 5.64. For this object, there are photometric data in 9 bands, corresponding to *χ*_*crit*_^2^ ≃ 15.51. Of all the objects considered, this is by far our best fit. Last, for JADES-GS-z11-0, our best fit is a 10^6^*M*_⊙_ SMDS formed via AC, at *z*_*photo*_ ≃ 11.66, delensed by a factor of *μ* ≃ 0.75.^**^ For this object, there are photometric data in 14 bands, and thus, this corresponds to a *χ*_*crit*_^2^ ≃ 22.36.

We note that in this paper, we have only considered a coarse grid of stellar masses. In the future, we should be able to find even better fits to the data by considering a finer grid of SMDS masses. The aim of the current paper is to point out that SMDS SEDs are generically excellent fits to JWST photometric data for Lyman break objects, not to find the best possible Dark Star fit to a given set of data.

#### Consistency with z_*spec*_.

We now compare our best fit redshifts *z*_*photo*_ obtained by fits of SMDS SEDs to JWST photometry, to the spectroscopic redshifts *z*_*spec*_ estimated by ref. 28, for each of the three candidate objects, in turn.

JADES-GS-z13-0: For this object *z*_*spec*_ ≃ 13.2, which is outside of our 95% CL (see the *Top Left* panel in Fig. 1). However, there are several reasons not to discard JADES-GS-z13-0 as a SMDS candidate. First of all, the estimation of *z*_*spec*_, given the *S*/*N* ≲ 2 of the spectra for JADES-GS-z13-0 (see the *Bottom Right* panel of Fig. 1 of ref. 28), has inherent uncertainties. Those will be significantly reduced once cleaner spectra are obtained.^††^ Furthermore, our *z*_*photo*_ will be slightly lower if we had a finer TLUSTY SED grid, with a slightly higher *M*_⋆_. Last, we have verified that *z*_*spec*_ ≃ 13.2 is within the 98% CL region, so statistically, our best fit is not ruled out at the 2 − *σ* level, even without accounting for the uncertainties explained above, that could significantly reduce this mild tension.

JADES-GS-z12-0: In this case *z*_*spec*_ ≃ 12.63, consistent with our *z*_*photo*_ ≃ 12.27, as it falls well inside the 95% CL (*Middle* panel of Fig. 1).

JADES-GS-z11-0: For this object, we find again excellent agreement between *z*_*spec*_ ≃ 11.58 and our best fit *z*_*photo*_ ≃ 11.66.

### Spectral Signatures of SMDSs Candidates.

In Fig. 1 (*Bottom* Row), we plot the photometric data (solid circles with error bars corresponding to the statistical uncertainty in each measurement) from ref. 27 against our SMDS models (red squares representing the average SMDSs flux in each band). The blue lines represent the redshifted (at *z*_*photo*_) best fit SMDS SEDs. Follow-up spectroscopy with high S/N could potentially identify the features in the SMDS spectra which would differentiate them from galaxies. A smoking gun, for all three SMDS candidates, is the He II *λ*1640 absorption line, which will either be absent in the case of galaxies without nebular emission or become an emission line for galaxies with strong nebular emission. Below, we discuss other spectral features for each candidate. The low S/N∼2 of the spectra obtained in ref. 28 does not conclusively identify any emission or absorption features, so in order to confirm their status as SMDSs, we would need follow-up, more detailed SEDs with NIRSpec (44) or other observatories.

To make a detailed comparison with spectra, we will also need to perform additional work on the modeling side. The TLUSTY spectra we have obtained need to also be run through CLOUDY (46) to take into account the effect of the nebula around the SMDS, in terms of both absorbing some of the light emitted by the SMDS as well as emitting light of its own. For the largest SMDS, which have accreted much of their surroundings, the nebular effects would be minimized. Consideration of the effect of the nebula on the spectra is the subject of future work.

JADES-GS-z13-0: For the 10^6^*M*_⊙_ SMDSs via capture modeling this object, we find that the spectrum includes a sequence of other He lines within the NIRSpec window; however, none of them are as strong as the aforementioned He II *λ*1640. In general, the hottest SMDSs, such as those formed via capture, will have a very mild Balmer break, if at all, and almost no absorption or emission features in the Balmer series. However, for those objects, the He II *λ*1640 is the strongest, in comparison to cooler SMDSs.

JADES-GS-z12-0: The 5 × 10^5^*M*_⊙_ SMDS via AC modeling this object has a nearly featureless SED, except for the absorption around 3.2 μm (redshifted). This corresponds to the He I *λ*3187 line. Also, this object, with a *T*_*eff*_ ≃ 1.7 × 10^4^ K, there is a rather pronounced Blamer break, at around 4.6 μm, with a jump in the flux of about 20% that should be detectable with NIRSpec.

JADES-GS-z11-0: The SED for the 10^6^*M*_⊙_ SMDSs via AC modeling this object has quite a few more He lines within the NIRSpec window, in comparison to the cooler 5 × 10^5^*M*_⊙_ discussed above. This is also the brightest of the three candidates, with a flux in many bands exceeding 15 nJy.

## Discussion and Conclusions

The purpose of this paper has been to show that SMDSs can generically provide good fits to JWST photometric data for high redshift (*z* ≳ 10) very luminous point-source candidates. Compared to the first galaxies, Dark Stars are significantly less complex, and as such, they are easier to model. Here, we considered the standard scenario of a single SMD that formed at the center of an early DM minihalo and is powered by DM heating. SMDSs are luminous and yet cool—far too cool for fusion to be taking place in their interiors. There are a set of undetermined parameters that control the formation and evolution of a Dark Star, and ultimately, its observable properties. In this paper, we assumed certain plausible values for DM particle parameters (we took the DM mass to be *m*_*χ*_ = 100 GeV and a canonical annihilation cross-section) and allowed four main astrophysical parameters to vary: two taking discrete values (mass of the SMDS, formation type of the SMDS) and two continuous parameters (redshift of emission and gravitational lensing factor) which we scanned over in order to find our best SMDSs fits to the photometric data. In the future, we plan to improve upon this analysis. In our previous papers that focused on building Dark Star models, we did consider a variety of DM masses, ranging from 1 GeV to 10 TeV. Since the heating rate scales as *σv*/*m*_*χ*_, studying a variety of particle masses is equivalent to studying a variety of annihilation cross-sections. We found that the existence of Dark Stars is generic, i.e., insensitive to these values. Yet a detailed comparison of SMDS models to JWST data will require a broader study of the possible DM particle input parameters. Further, in the future, we plan to use Bayesian statistics to infer posterior distributions (i.e., best fits) for DM as well as astrophysical parameters. However, at this stage, this type of analysis is premature, as we will need high-quality spectra for this purpose.

In conclusion, in this work, we have identified three SMDS candidates at *z* ∈ [11, 14] in the JWST data: JADES-GS-z13-0, JADES-GS-z12-0, and JADES-GS-z11-0. For each of them, a low redshift contaminant is excluded in view of the spectroscopic detection of the Lyman break (28). Additionally, we made predictions for the spectra of those SMDS candidates and suggested smoking gun signatures such as the He II *λ*1640 absorption line, a feature expected for all SMDSs but not for Pop III/II galaxies. We further note that the spectra of SMDS and early galaxies differ for wavelengths above ∼5 μm, so that future observatories (beyond JWST) might be able to differentiate the two types of objects in this way. The three JADES objects are currently consistent with point objects given the limitations of the angular resolution of JWST; in the future, if Airy patterns were identified for any SMDS candidate, that might confirm its point-like nature. The confirmation of even a single one of those objects as a Dark Star (with detailed NIRSpec spectra) would mark a new era in astronomy: the observational study of DM–powered stars.

## Supplementary Material

Appendix 01 (PDF)

## Data Availability

There are no data underlying this work.

## Footnotes

^*^In ref. 30, one of us studied a concrete SIDM model that powers DS via annihilation and found that SIDM-powered Dark Stars have the same properties, such as their luminosity, size, and hence observable signatures in JWST, as Dark Stars predicted in WIMP DM models.

^†^We have confirmed the results of the simple Blumenthal et al. (31) AC approach via more sophisticated analyses (32).

^‡^In other words, the objects are detected in one redshift bin but not in a neighboring one, due to absorption by Ly-α along the line of sight. The wavelength of the observed drop-off in flux (redshifted from the time of the emission of the light) is then used to identify the redshift of the object.

^§^Thus, we avoid potential low redshift interlopers as another possible interpretation of the photometric data. Dropping condition i) would allow us to find more SMDS candidates, and we plan to do so in a future publication.

^¶^Those concentric bulls-eye shaped patterns arise due to diffraction off of the aperture of the telescope and are unique to point sources.

^#^This assumes, to a first-order approximation, a circular aperture. The actual point spread function of JWST is more complex; see ref. 38 for details. However, the main conclusions regarding Airy patterns are unaffected by this complication.

^ǁ^As commonly denoted, *z_photo_* represents the best fit value of the emission redshift for an object, in view of available photometric data (i.e., average fluxes over various bands) and theoretical models for SEDs.

^**^If we refined our SMDS mass grid, we would be able to find an equally good or better fit with slightly lower *M*_*_ and μ ≳ 1. Yet at high redshifts, typical objects have a μ < 1 (see figure 4 of ref. 42).

^††^For instance, for GN-z11, *z_spec_* ⋍ 11:1 from HST Grism (43), whereas the exquisite spectrum obtained recently with JWST using NIRSpec (44) by Bunker et al. (45) leads to a more accurate *z_spec_* ⋍ 10:60.
