# Supplementary material for: Supermassive Dark Star candidates seen by JWST
Source: Proc Natl Acad Sci U S A. 2023 Jul 11;120(30):e2305762120. doi: 10.1073/pnas.2305762120 (PMC10372643; doi:10.1073/pnas.2305762120)
Supplement: Supplementary file 1 — Appendix 01 (PDF) [file pnas.2305762120.sapp.pdf]

Supporting Information for

Supermassive Dark Star candidates seen by JWST

Cosmin Ilie, Jillian Paulin and Katherine Freese

Corresponding Katherine Freese.
E-mail: ktfreese@utexas.edu

This PDF file includes:

Supporting text
Figs. S1 to S5
Tables S1 to S2
SI References

Supporting Information Text

Note. The Supplementary Material contains additional details relevant to our work presented in the main text. Additionally, for the reader's convenience, it presents enlarged versions of our plots included in the main text, which, due to page limits, were combined into the six panel Fig. 1 in the main text. This Supplementary Material is divided into two sections: Dark Star Spectra and HR Diagram, and SMDS Candidates.

1. Dark Star Spectra and HR Diagram

In Table S1 we list the relevant parameters for SMDSs on our stellar mass and formation mechanism grid that were then passed to TLUSTY to obtain relevant SEDs. The values listed here are adopted from (1) (see Tables 3 and 4 there). We assume both types of SMDSs are powered by annihilations of 100 GeV WIMPs and formed in $10^8 M_\odot$ DM halos at redshift $z_{form} = 15$ and grow via accretion, at a rate of $\dot{M} = 10^{-1} M_\odot \mathrm{yr}^{-1}$. For the case of a SMDS formed via DM capture, we further assume that the product between the ambient DM density and the DM-proton scattering cross section is: $\rho_\chi \sigma = 10^{14}$ GeVcm$^{-3} \times 10^{-40}$cm^2.

Table S1. Parameters of SMDSs of various masses if formed via Extended AC or DM Capture.

Formation Mechanism	M_* ($M_\odot$)	L_* ($10^6 L_\odot$)	R_* (AU)	$T_{\rm eff}$ (10^3 K)
Extended AC	2.04×10^4	407	31	10
Extended AC	10^5	2.42×10^3	39	14
Extended AC	5×10^5	7.21×10^3	46	17
Extended AC	10^6	2.01×10^4	61	19
Capture	4.1×10^4	774	1.8	49
Capture	10^5	1.75×10^3	2.7	51
Capture	10^6	2.03×10^4	8.5	51

In Fig. S1 we plot the restframe SEDs of SMDSs of various masses (with values labeled in the legend) formed via the two mechanisms described in the main text: Adiabatic Contraction and DM Capture. The parameters for each star are given in the caption, and were obtained in (1), when two of us used the polytropic approximation to model SMDSs. We note here that for radiation pressure dominated stars, such as SMDSs, a polytrope of index $n = 3$ is an excellent approximation.

In Fig. S2 we present the HR diagram for Dark Stars (reproduced from (1)). Sold lines represent the evolutionary tracks of SMDSs formed via DM Capture (for three different WIMP masses), whereas the set of three lines to the right (dotted, dashed, and dash-doted) represent the evolutionary tracks of SMDSs formed via extended-AC (for the same three values of the WIMP masses, as labeled in legend). All objects start from the lower right corner, i.e. cool and dim. They grow via accretion to the stellar masses indicated.

For more details on Dark Stars and their observability please see the following review:(2).

2. SMDS Candidates in JWST

We start this section with Table S2, where we list the best-fit parameters for each SMDS candidate as a match to objects in JWST data as labeled: $z_{\rm phot}$ is the photometric redshift assuming the SMDS mass and formation mechanism listed in the last two columns of this table. $z_{\rm spec}$ is the spectroscopic redshift found in (3). μ is the gravitational lensing factor for our SMDSs best fits. χ^2 is the value found during our analysis; $\chi^2_{\rm crit}$ is the value of χ^2 required for 95% confidence in our result; and $\chi^2_{\rm gal}$ is the value of χ^2 found assuming these objects are galaxies, as in (3).

Table S2. The best-fit parameters corresponding to each of the SMDS candidates.

Candidate	$z_{\rm phot}$	$z_{\rm spec}$	μ	χ^2	$\chi^2_{\rm crit}$	$\chi^2_{\rm gal}$	Formation Mechanism	SMDS Mass ($M_\odot$)
JADES-GS-z13-0	13.98	13.20	1.50	14.12	18.3	6.8	Capture	10^6
JADES-GS-z12-0	12.27	12.63	1.11	5.64	15.5	3.6	Extended AC	5×10^5
JADES-GS-z11-0	11.66	11.58	0.75	12.23	22.4	14.7	Extended AC	10^6

In this section we additionally present enlarged versions of the plots included in Fig. 1 from the main text, as follows: JADES-GS-z13-0 , in Fig. S3, JADES-GS-z12-0 in Fig. S4, and finally JADES-GS-z11-0 in Fig. S5.

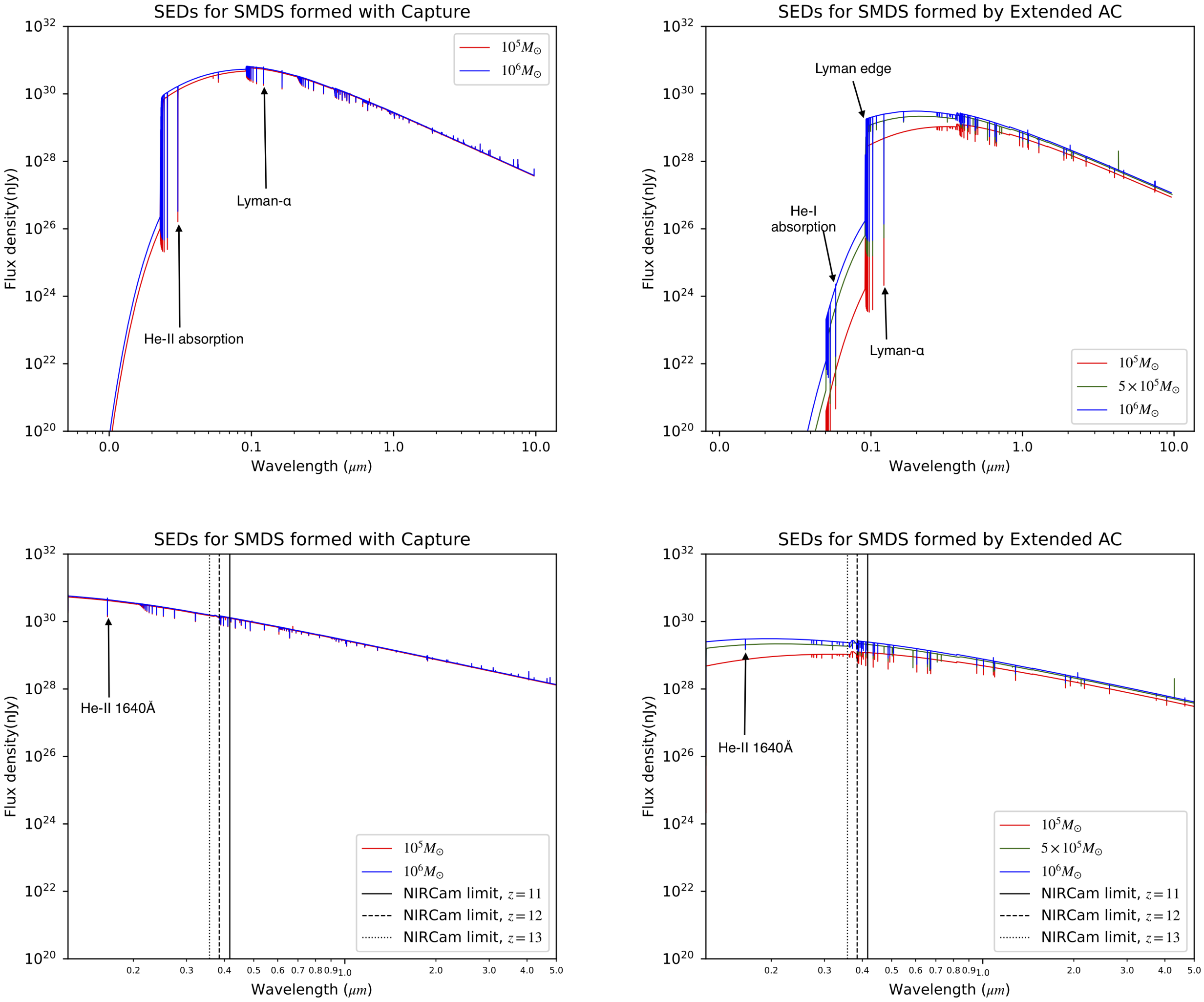

Fig. S1. TLUSTY simulated SEDs of supermassive dark stars of different masses. The left panels represent SMDS formed with capture and the right panels represent SMDS formed via extended AC. Parameters for these model SMDS can be found in table S1. In the lower panels, we zoom into the region observable by NIRCam and NIRSpec, between Ly-α and $5\mu m$. The NIRCam limits (corresponding to $\lambda_{obs} = 5\mu m$) at different redshifts are shown in black. At the other end, we are limited by the Gunn-Peterson trough. Therefore, NIRCam/NIRSpec will only observe the very narrow part of the SED between Ly-α and the vertical black lines.

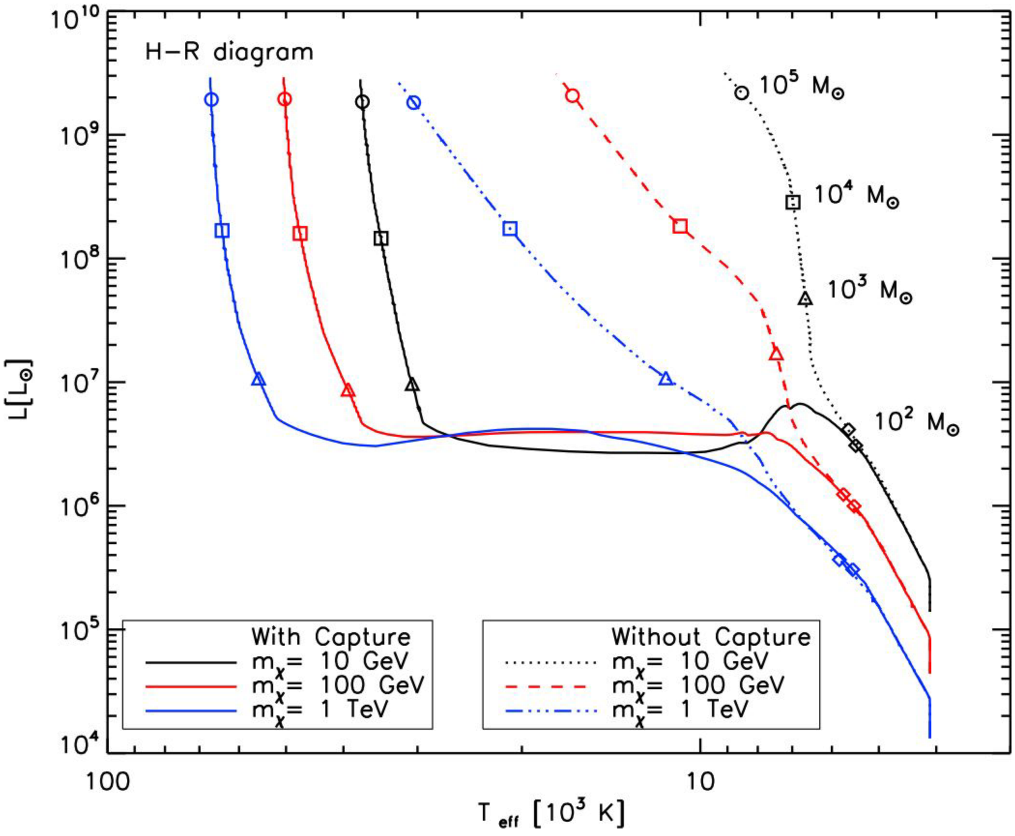

Fig. S2. Hertzsprung-Russell (HR) diagram for dark stars for accretion rate $\dot{M} = 10^{-3} M_\odot$/yr and a variety of WIMP masses as labeled for the two cases: (i) "without capture" but with extended adiabatic contraction (dotted lines) and (ii) "with capture" (solid lines). The case with capture is for product of scattering cross section times ambient WIMP density $\sigma\bar{\rho}_\chi = 10^{-40}\mathrm{cm}^2 \times 10^{14}$GeV/cm^3. Also labeled are dark star masses. The final DS mass was taken to be $1.5 \times 10^5 M_\odot$ (the baryonic mass inside an assumed $10^6 M_\odot$ DM host halo), but it could be larger, depending on the mass of the host halo as well as the evolution of the SMDS inside merging minihaloes. (Figure reproduced from (1)).

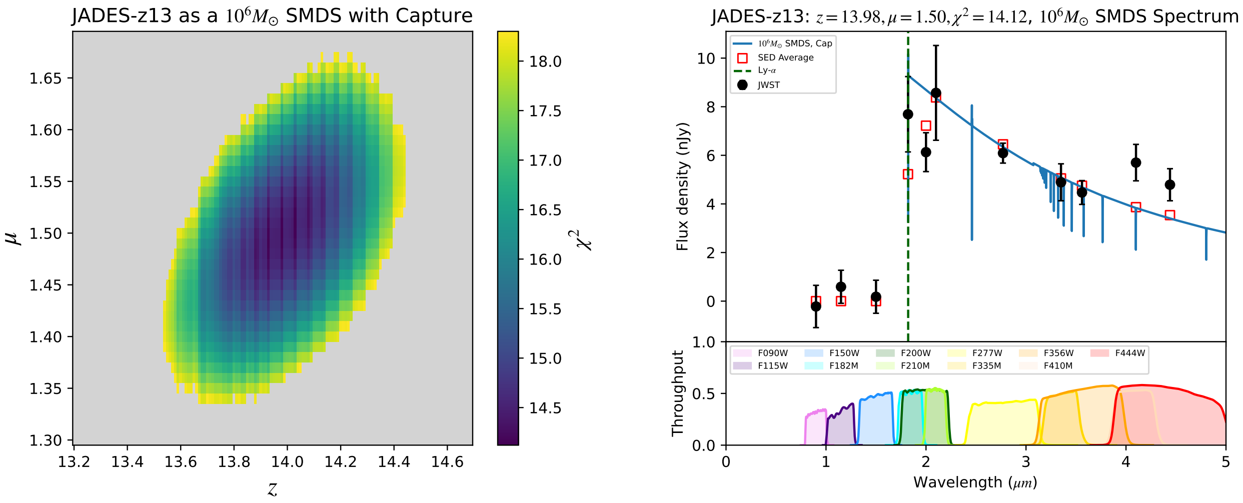

Fig. S3. (Left Panel) Optimal fit region in the z vs μ parameter space for JADES-GS-z13-0 as a $10^6 M_\odot$ SMDS formed via DM capture. The heatmap is color coded according to the value of the χ^2, and is cut off (grayed out) at the critical value corresponding to 95% CL. (Left panel) We plot our best fit SMDSs SEDs against the photometric data of (3) in each band (color coded and labeled in legend). For a $10^6 M_\odot$ SMDSs formed via DM capture, our best fit parameters take the following values: $z_{photo} = 13.98$ and $\mu = 1.50$. For our best fit we have $\chi^2 = 14.12$, whereas the critical value, corresponding to 95% CL is, given the 11 bands in which we have data, $\chi_{crit} = 18.3$.

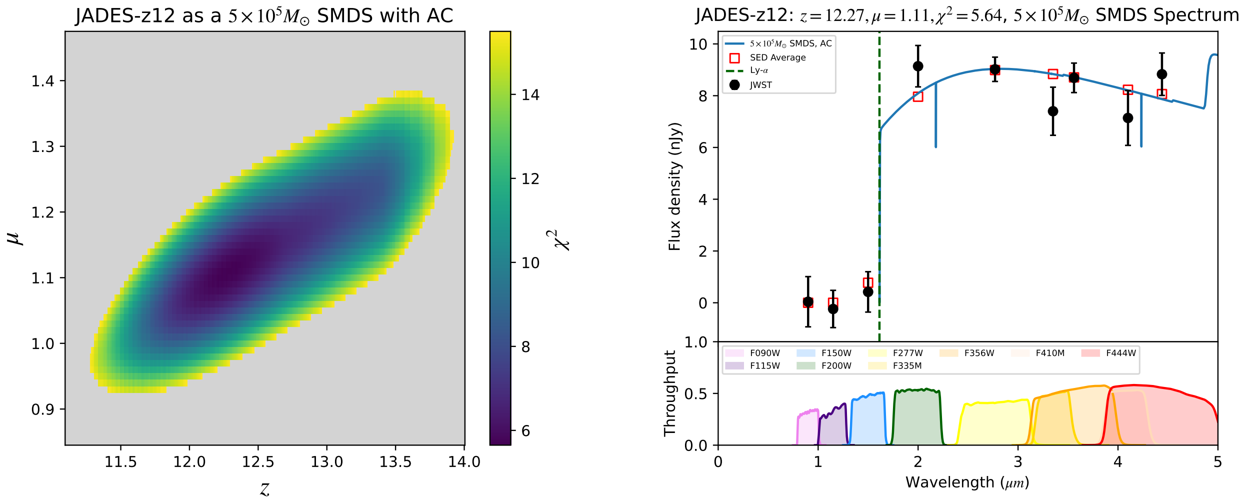

Fig. S4. (Left Panel) Optimal fit region in the z vs μ parameter space for JADES-GS-z12-0 as a $5 \times 10^5 M_\odot$ SMDS formed via AC. The heatmap is color coded according to the value of the χ^2, and is cut off (grayed out) at the critical value corresponding to 95% CL. (Left panel) We plot our best fit SMDSs SEDs against the photometric data of (3) in each band (color coded and labeled in legend). For a $5 \times 10^5 M_\odot$ SMDSs formed via AC, our best fit parameters take the following values: $z_{photo} = 12.27$ and $\mu = 1.11$. For our best fit we have $\chi^2 = 5.64$, whereas the critical value, corresponding to 95% CL is, given the 9 bands in which we have data, $\chi_{crit} = 15.51$.

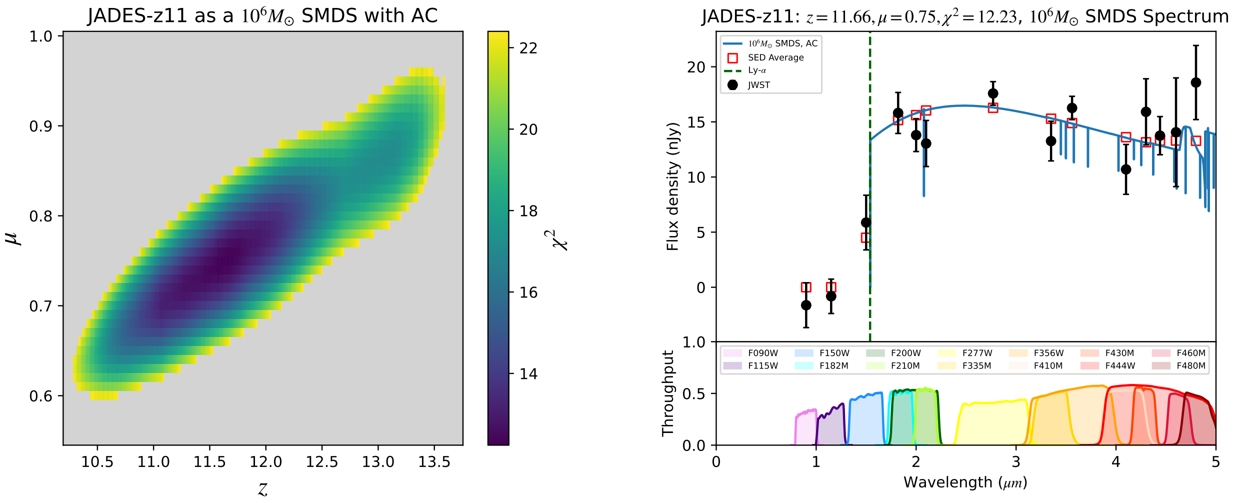

Fig. S5. (Left Panel) Optimal fit region in the z vs μ parameter space for JADES-GS-z11-0 as a $10^6 M_\odot$ SMDS formed via AC. The heatmap is color coded according to the value of the χ^2, and is cut off (grayed out) at the critical value corresponding to 95% CL. (Right panel) We plot our best fit SMDSs SEDs against the photometric data of (3) in each band (color coded and labeled in legend). For a $10^6 M_\odot$ SMDSs formed via AC, our best fit parameters take the following values: $z_{photo} = 11.66$ and $\mu = 0.75$. For our best fit we have $\chi^2 = 12.23$, whereas the critical value, corresponding to 95% CL is, given the number of bands in which we have data, $\chi_{crit} = 22.36$.

References

1. K Freese, C Ilie, D Spolyar, M Valluri, P Bodenheimer, Supermassive Dark Stars: Detectable in JWST. *Astrophys. J.* **716**, 1397–1407 (2010).
2. K Freese, T Rindler-Daller, D Spolyar, M Valluri, Dark stars: a review. *Reports on Prog. Phys.* **79**, 066902 (2016).
3. BE Robertson, et al., Discovery and properties of the earliest galaxies with confirmed distances. *arXiv e-prints* p. arXiv:2212.04480 (2022).
